# Merging the Religious Congregations and Membership Studies: A Data File for Documenting American Religious Change

**DOI:** 10.1007/s13644-018-0339-4

**Published:** 2018-05-24

**Authors:** Rachel Bacon, Roger Finke, Dale Jones

**Affiliations:** 1 The Pennsylvania State University, 211 Oswald Tower, University Park, PA 16802 USA; 2 Church of the Nazarene Global Ministry Center, 17001 Prairie Star Parkway, Lenexa, KS 66220 USA

**Keywords:** Religion census, Religious change, Data, Longitudinal analyses

## Abstract

The decennial religious congregations and membership studies are a popular data source for analyzing local religious composition and diversity, but several methodological challenges hinder merging the datasets for longitudinal analyses. In this paper, we introduce strategies for addressing four of the most serious challenges: religious mergers and schisms, changes in membership standards within certain groups, missing data and changes in county boundaries. In doing so we successfully merge the 1980, 1990, 2000 and 2010 collections and build new longitudinal datasets of congregational and membership counts at the state and county levels. These changes increase religious group representation from 48 to 76, reduce bias from missing data, allow for the more reliable inclusion of 20–23 million adherents in each year, and improve overall ease of use. We also document instances when corrections were not possible and alert readers to the limitations of the merged files when measuring change among certain groups. The new longitudinal files are accessible from theARDA.com.

The decennial religious congregations and membership studies collected by the Association of Statisticians of American Religious Bodies are perhaps the most heavily used datasets on American religion. These data provide counts of adherents and congregations belonging to dozens of religious groups at the state and county levels. Scholars of varied backgrounds rely on these data to measure and analyze local religious composition, diversity, and the contextual effects of religion (Lim 2013). The four most recent of these datasets (i.e. 1980, 1990, 2000, 2010) have been collectively downloaded more than 60,000 times from the Association of Religion Data Archives (theARDA.com) over the past 10 years. Moreover, religious denominations and local congregations, as well as the media and community planners, use the files extensively for research, journalism, and planning. In short, the collections have proven invaluable to a diverse group of users for mapping and analyzing religious variations across counties, states and the nation.

Despite the popularity of these collections, they are seldom used to measure *changes* in religious group size or the composition and diversity of geographic regions overtime. Several practical and methodological challenges deter researchers from merging the datasets. This paper introduces methodological strategies to address four of the most significant challenges for merging the collections: religious mergers and schisms, changes in membership standards within certain groups, missing data, and changes in county boundaries. Relying on these strategies we merge the 1980, 1990, 2000 and 2010 collections and build two datasets of congregational and membership data across time: one for counties and another for states.^1^ When possible, we make corrections or offer improved estimates to standardize measures and units of analysis over time. When corrections are not possible, however, we alert readers to the limitations of the merged files.

Before we address the challenges of merging the data collections, however, we begin with a brief overview of the data collection procedures used. Documenting how the data are collected helps to clarify and identify the challenges that emerge when we attempt to merge the collections.

## Collecting Congregational and Membership Data

The congregational and membership studies conducted in 1980, 1990, 2000 and 2010 each provide a county-by-county enumeration of religious bodies in the U.S.. Although similar collections also were conducted in 1952 and 1971 (National Council of Churches 1956; Johnson et al. 1974), we have not included them because the level of participation was lower and the measures used were less standardized than the later collections. The remaining four datasets relied on procedures and measurement criteria that were largely standardized. Nevertheless, we highlight several important differences between the remaining four collections.

In each of the decennial studies, a study representative contacted all of the major religious groups and asked them to report on the number of congregations, members and adherents in their religious group for each county in the U.S.. We focus on congregational counts and total adherents because these measures are more consistently reported across religious groups and are more standardized than membership (membership counts are also unavailable in the online versions of the 2000 and 2010 datasets). For many groups, especially Christian groups practicing adult baptism, full membership status was reserved for adults. In contrast, adherents is a more inclusive measure defined as “all members, including full members, their children and the estimated number of other participants who are not considered members” (Quinn et al. 1982). Not all religious groups directly reported both membership and adherent data. When religious groups reported only adult members, the data collectors estimated the number of total adherents using a county-level multiplier.^2^ Most religious groups who directly reported adherents agreed to the standard definition, but several groups changed their counting methodology over the years, usually to make their adherent counts more accurate and more comparable to other groups. Data collectors also commissioned independent studies for groups that were difficult to count accurately, such as the Amish and most non-Christian groups. These independent studies were not always conducted by the same principal investigators (PIs) from one decade to the next and employed different methodologies. Among those groups affected by changes in measurement, therefore, counts differ substantially between the datasets and may not reliably reflect changes in size.

We also call attention to the fact that the scope of the data collections has expanded over time, resulting in varying levels of data collection efforts allocated to specific groups. For the 1980 and 1990 collections the PIs appealed only to Christian denominations and Jewish groups, capturing the vast majority of white Americans attending local congregations. Later collections expanded to include independent Christian congregations as well as non-Christian groups. Despite the PIs’ efforts to include as many groups as possible, the level of participation varied across the datasets, with 109^3^ participating in 1980, 133 in 1990, 149 in 2000, and 236 in 2010. As shown in greater detail in online supplement Appendix A (all appendices are available at theARDA.com), however, only 48 of the cumulative 302 participating groups have both adherent and congregation data in all four decades. The considerable variation in participation can be partly explained by the expanded scope of the collection procedures in later decades. Furthermore, some groups merged, split, or simply reported their data differently from one decade to the next, while other groups simply participated in some years, but not others. The result is that most groups have missing data for at least 1 year.

Finally, we should note that the official name of the datasets and the sponsoring institutions have varied from year to year. The 1980 collection was assembled by the Glenmary Research Center and the final three collections were conducted by the Association of Statisticians of American Religious Bodies.^4^ The 1980 and 1990 datasets were entitled “Church and Church Membership in the United States,” reflecting their focus on Christian groups. The 2000 dataset was changed to “Religious Congregations and Membership Study” and the 2010 dataset was revised to the “U.S. Religion Census: Religious Congregations and Membership Study.” To reduce confusion, throughout this paper we refer to the four datasets as the religion census data, and the final dataset as the merged or longitudinal file.

The religion census data offer great promise for conducting longitudinal and spatial analyses on religious change in America, but merging the files and offering comparable data over time poses multiple challenges. Below we review these challenges, offer recommendations on how some can be addressed and review the limitations that will remain.

## Addressing the Challenges

In this paper, we address four major challenges in merging the four religion census datasets. First, we devise a new scheme to address inconsistency in variable names across the datasets because of mergers, schisms or changes in how a group’s data were aggregated over time. Second, we document how some groups changed their standards for reporting adherent and/or congregation counts between collections and we clearly delineate between the counts that can be rectified and those that cannot. Third, we reduce the amount of missing data by offering adherent estimates for groups missing adherent and/or congregation data for a single year and by providing comparable estimates overtime for groups involved in mergers or schisms. Fourth, we account for changes in the geographic boundaries of counties, allowing for a comparable unit of analysis over time. In addressing the problems, we produce a merged dataset of the four collections that readily permit trend analysis of adherent and congregation counts, although with some caveats that we clearly identify.

### Accounting for Mergers, Schisms, and Other Aggregations

Religious group schisms occur when a group splits into two or more groups, and a merger occurs when two or more religious groups become one group, which makes it difficult to consistently identify the same group of adherents over time. This problem is further compounded by measurement errors, such as accidentally omitting one of the religious groups involved in the merger, schism or some other aggregate group change. Since religious groups are geographically concentrated and therefore non-randomly distributed (Land et al. 1991; Stump 1998; Finke and Stark 2005), the unintentional omission of a group in longitudinal analyses could introduce significant bias. We address both of these problems in the new longitudinal file.

We begin by replacing the old naming scheme for variables with one that is more consistent across the data collections. In the stand-alone versions of the religion census data available online, the variable names assigned to individual religious groups are different in each dataset. Data are presented in “wide” format (i.e. each row is a county and the dozens of columns contain the counts of each religious group’s adherents and congregations separately) and users must rely on the name of the religious group to match them across datasets. Although most groups retain the same name for each year they participated, their name can change due to mergers, splits or changes in how the data were reported. In the case of schisms, adherents who previously belonged to one religious group are split among two or more groups after the schism. In mergers, the opposite occurs. In addition, some groups in the religion census data did not formerly split or merge, but agreed to report together in 1 year and separately in another.

To alleviate the burden of identifying groups across the datasets for users, we first replaced the old variable names with the unique three-number codes assigned to each religious group by the religion census data collectors. Second, we created new aggregate cases that combine all groups affected by a schism, merger, or other group count change into a single group. The full listing of religious group names and their accompanying codes are in Appendix A and the “notes” column identifies which groups are combined on account of a merge, split or change in aggregated reporting. Our solution resulted in eight new aggregate groups created from 27 individual religious groups:

- A total of five groups present in the religion census data were affected by a schism and were combined into two new groups for the longitudinal file.
- Six groups experienced a formal merger and were combined into two new groups.
- Sixteen groups chose to report together in 1 year but separately in another, and we subsequently combined them into four new groups.

Table 1 identifies the affected religious groups and their sizes. Newly combined groups are now assigned the three-digit code that matches the largest group among them, followed by the letter “b” to indicate that multiple groups were combined to make the new group. Data in the merged file are also presented in “long” format (i.e. rows now represent religious groups in a specific county and year, and only two columns are used to identify adherent and congregation counts). Groups that would otherwise be dropped because they do not appear to participate across the datasets are now included, resulting in nearly two million additional adherents across all four datasets. In this way, the longitudinal file includes both the combined groups and the individual counts of each religious group that was included in the combined group.^5^

**Table 1:** Adherents of groups affected by schisms, mergers, or other changes in aggregation

	Code	1980	1990	2000	2010
*Schisms*					
Episcopal Church groups combined	193b	2,823,399	2,445,286	2,314,756	2,085,445
Episcopal Church	193	2,823,399	2,445,286	2,314,756	1,951,907
The Anglican Church in North America	033				133,538^a^
Evangelical Lutheran Church in America Groups Combined	207b	5,379,943	5,226,798	5,113,418	4,287,768
Evangelical Lutheran Church in America	207	5,379,943	5,226,798	5,113,418	4,181,219
Lutheran Congregations in Mission for Christ	255				310,185
North American Lutheran Church	314				106,549^a^
*Merges*					
Mennonite Groups Combined	288b	165,164	195,210	171,210	141,647
Conservative Mennonite Conference	183			14,865	14,284
Mennonite Church	285	118,273	154,259		
The Mennonite Church General Conference	287	46,891	40,951		
Mennonite Church USA	288			156,345	127,363
Serbian Orthodox Church in the USA Groups Combined	410b			83,520	68,760
Serbian Orthodox Church in the USA	410			55,807	68,760
Serbian Orthodox Church in the USA (New Gracanica Metropolitanate)	411			27,713^a^	
*Changes in aggregate reporting*					
Friends Groups Combined	226b	137,401	130,484	113,086	104,609
Friends (Quakers)	226	137,401	130,484	113,086	
Friends Central Yearly Meeting	085				290
Friends United Meeting	225				24,826
Friends—Evangelical	328				34,565
Friends—Independent Yearly Meetings	338				2850
Friends—Unaffiliated Local Meetings	389				2474^a^
Friends—Conservative	390				1976
Friends—General Conference	391				22,192
Friends—Dual General Conference and United Meeting	393				15,436
Bruderhof and Hutterian Groups Combined	257b		11,037	13,224	15,073
Hutterian Brethren	257		11,037	12,300	13,260^a^
Bruderhof Communities, Inc.	070			924	1813
Orthodox Church in America Groups Combined	331b			108,877	84,928
Orthodox Church in America: Territorial Diocese	331			77,110	84,928
Orthodox Church in America: Albanian Orthodox Archdiocese	007			5775	
Orthodox Church in America: Romanian Orthodox Episcopate of America	397			17,201	
Orthodox Church in America: Bulgarian Diocese	332			8791	
Moravian Church in America, Alaska and North Province Combined	293b	36,961	36,588	28,434	22,935
Moravian Church in America, Alaska province	292		5338	2562	2649
Moravian Church in America (Unitas Fratrum) North Province	293	36,961	31,250	25,872	20,286
Total Adherents Added After Adjustments		302,565	342,069	492,479	657,753

^a^Adherent count is estimated for these groups

Once we identified the groups that needed to be combined, however, some data remained missing. In five instances, one of the groups included in a combined group was missing adherent counts in 1 year, so we estimated the missing adherent counts in these cases (please see the section on missing data for methodological details). Because schisms are often regionally based, this correction reduces the geographic bias that would exist if they were omitted. In other cases, a few of the schismatic groups simply did not participate. For example, the Alliance of Baptists (AOB) split from the Southern Baptist Convention (SBC) in 1987 and is included in the 2010 religion census collection. We chose not to combine the 2010 AOB counts with the SBC because the AOB only provided data on the number of congregations in 2010 and provided no data at all in 1990 and 2000.^6^ Individual congregations also are known to switch affiliations or become independent churches at a smaller scale than formal schism (Marcum 2017; Chaves 2017), making it impossible to track the adherents of congregations who leave the denomination to another group in the data. Most of these omissions, however, are small and will have little impact on the total counts. Overall, our adjustments for known schisms, mergers and other aggregations allow for meaningful comparisons over time for more groups and greatly ease the use of the longitudinal files.

### Group Measurement Changes

A second major challenge for constructing a longitudinal file is when some groups change the criteria they use for counting adherents or congregations from one collection to the next. As noted earlier, the criteria or methods used for collecting the data were sometimes changed in an effort to improve the counts or make their measurement comparable to other groups in the collection. By making the change, however, the adherent or congregation counts for the group were no longer comparable across all four collections. This is a significant concern because it affects four of the 10 largest religious groups in the country and because it is often the most difficult to correct.

Table 2 lists all of the groups where measurement criteria or methods changed for at least one time point, reports on the size of the group, and offers recommendations on when over time comparisons can be made. Below we offer additional information and additional precautions for each of these groups.^7^ Before we review these details, however, we first explain how we were able to offer corrections for the measurement changes of the United Methodist Church.

**Table 2:** Groups with measurement changes and recommendations for comparisons

	1980	1990	2000	2010	Possible over-time comparisons
*Catholic*					
Adherents	47,502,152	53,385,998	62,035,042	58,927,887	1980, 1990, 2000; caution when using 2010
Congregations	22,348	22,441	21,791	20,589	All years
Groups	1	1	1	1	Same group is measured in all years
*United Methodist*					
Adherents (Old)	11,552,111	11,091,032	10,350,629	9,275,866	All years
Adherents (New)	–	–	11,035,532	9,860,653	2000, 2010
Congregations	38,465	37,238	35,721	33,323	All years
Groups	1	1	1	1	Same group is measured in all years
*Latter-Day Saints*					
Adherents	2,684,744	3,540,820	4,224,026	6,144,582	1980, 1990, 2000; caution when using 2010
Congregations	6,771	9,208	11,515	13,601	All years
Groups	1	1	1	1	Same group is measured in all years
*Southern Baptists*					
Adherents	16,281,692	18,940,682	19,881,467	19,896,279	All years
Congregations	35,552	37,922	41,514	50,816	1980, 1990, 2000; caution when using 2010
Groups	1	1	1	1	Same group is measured in all years
*Independent Churches*					
Adherents	–	2,001,427	2,051,937	12,241,329	1990, 2000; do not compare with 2010
Congregations	–	2192	1705	35,496	1990, 2000; do not compare with 2010
Groups	–	2	2	1	1990, 2000 Charismatic/non-Charismatic
*Black Protestants*					
Adherents	1,778,735	9,888,728	–	4,877,067	No years definitively comparable
Congregations	3634	2017	–	17,754	No years definitively comparable
Groups	4	4	–	12	No years definitively comparable
*Jewish Groups*					
Adherents	802,726	5,982,529	6,141,325	2,256,584	1990, 2000; do not compare with 1980 or 2010
Congregations	1501	3975	3727	3464	1990, 2000; do not compare with 1980 or 2010
Groups	1	1	1	4	Jewish count split into four groups for 2010
*Old Order Amish*					
Adherents	85,676	121,750	96,986	251,808	No years definitively comparable
Congregations	535	830	1290	1839	No years definitively comparable
Groups	1	1	2	1	Additional Amish group reported with the Old Order Amish in 2010, but separately in 2000
*Orthodox Groups*					
Adherents	54,849	152,556	1,082,730	1,056,535	2000, 2010; do not compare with 1980 or 1990
Congregations	71	1163	2053	2551	2000, 2010; do not compare with 1980 or 1990
Groups	4	11	25	27	Same 23 groups present in 2000 and 2010
*The Wesleyan Church*					
Adherents	–	259,740	381,459	250,051	No years definitively comparable.
Congregations	–	1657	1657	1614	No years definitively comparable
Groups	–	1	1	1	Same group is measured in all years

For the United Methodist Church (UMC), there was a change in adherent measurement between 2000 and 2010. Prior to the 2010 collection, the UMC reported membership, but not adherent counts. Because adherents were not directly reported, the religion census data collectors estimated adherents using the membership count and their standard county-level multiplier (see footnote 2). In 2010, however, UMC statisticians directly reported their own adherent totals for each congregation, and this shift yielded more adherents than the old estimate. The county-level estimation would have identified 9.3 million adherents in 2010 rather than the 9.9 million reported by UMC statisticians (see Table 2). Since the UMC submitted both membership and adherent counts to the religion census in 2010, it is possible to use the old county-level estimation formula to create an adherent count consistent with earlier datasets. We also requested from the UMC statisticians a direct reporting of adherent counts for 2000, which made it possible to use the direct-reporting measurement for 2000 and 2010. Both the “new” and “old” versions of UMC adherents for 2000 and 2010 are included in the longitudinal file.

In the case of the UMC, the measurement change was relatively simple and we were able to standardize the 2010 adherent count with earlier years using membership and a county-level multiplier. Other groups have more complicated changes and we were unable to standardize their counts across all four datasets. Like the UMC, the measurement of Roman Catholic Church adherents switched to a more direct-reported adherent count in 2010. Unlike the UMC, however, earlier counts relied on some survey-based estimates, which could not be standardized with the new count method. The Southern Baptist Convention and Church of Latter-day Saints changed their definition of congregations or adherents and, unlike the UMC, were unable to provide counts to us based on their old/new definition. For Eastern Orthodox groups and the Wesleyan Church, we are unable to determine the methodology used in some years, which calls into question measurement consistency across time. Among the remaining cases of measurement change, religion census data collectors commissioned independent studies to improve the accuracy of measurement. Differences in data collection methods and strategies used by these independent studies contribute another source of measurement inconstancy over time. Each group has a unique measurement history that limits comparisons between two or more collection years. We describe these histories in more detail here:

- *The Catholic Church* In 2000, some adherent counts were available at the parish-level, but in many areas the data collectors relied on diocese-level vital and sacramental statistics or on county-level estimates made using the American Religious Identification Survey (Jones et al. 2002). The shift to congregational-based direct reports in 2010 greatly reduced the number of Catholic adherents. The number of Catholics appears to decline from 62.0 million in 2000 to 58.9 million in 2010, but the figure reported to the Yearbook of American and Canadian Churches (YACC) suggests an increase from 63.7 to 68.2 million over the same time period (Lindner 2000, 2010). Some survey estimates of Catholics do identify a recent flattening in adherents, but most do not (Grammich 2012), which suggests the decline captured in the religion census is a data artifact.
- *Church of Latter-day Saints (LDS)* For the 2000 collection, the LDS Church reported a total of 4.2 million adherents. This total excluded members who were baptized, but not associated with a specific congregation. A more inclusive count is the 5.2 million members reported by the Church in its Almanac and to the YACC (Lindner 2000). For the 2010 collection, the LDS Church changed its procedures to include the previously excluded baptized persons and reported an adherent total of 6.1 million, a figure more consistent with what it has reported elsewhere (Lindner 2010) and more comparable to how other denominations count adherents in the religion census.
- *The Southern Baptist Convention (SBC)* The SBC reported adherents in a consistent way over time, but changed how they defined congregations. Prior to 2010, the congregation count did not include small “mission” gatherings, although adherents attending “missions” were counted. The SBC revised their 2010 methodology to include their mission congregations and therefore became consistent with other religious groups (Grammich et al. 2012). As a result, there is a sudden increase of 9000 SBC congregations between 2000 and 2010 despite negligible growth in the number of adherents.
- *Eastern Orthodox groups* In 1980 and 1990, four Orthodox groups directly reported adherent counts and an additional seven groups provided congregational counts for 1990, but they provided no documentation regarding counting methodology (Quinn et al. 1982; Bradley et al. 1992). For the 2000 and 2010 collections, however, data on a larger number of Eastern Orthodox groups were collected by a single PI using a more standardized definition of “adherents that included both adults and children known to participate in services at the local parish” (Jones et al. 2002). As a result, the counts for several Orthodox groups change suddenly between 1990 and 2000.
- *The Wesleyan Church* The documentation of data collection methodologies are available for 2000 and 2010, but not for 1990, and a significant change in estimation is suggested between decades. In 2000, the Wesleyan Church provided adherent counts based on their own record-keeping, reflecting “those who have some contact with the church through its various departments” (Jones et al. 2002). In 2010, they estimated that adherents were simply 30 percent more than the average attendance (Grammich et al. 2012).
- *Black Protestant groups* Only four historically Black Protestant groups directly reported their own counts in the 1980 collection (Quinn et al. 1982). In 1990, three Black Protestant groups participated and an independent study estimated the size of Black Baptist Churches, but the estimates did not include any congregational counts (Bradley et al. 1992). Black Protestant groups are entirely absent in the 2000 collection, although Finke and Scheitle (2005) did create a state-level multiplier based on survey data to estimate the size of all Black Protestant adherents for that year. Relying on congregational estimates from the online Infogroup database on religious organizations, the 2010 collection included membership estimates for eight Black Protestant denominations and congregational estimates for 12. Adherent counts were not available for all Black Protestant congregations, and so data collectors estimated a membership of 100 for those congregations (Grammich et al. 2012).
- *Jewish groups* The 1980 collection included direct counts of Conservative and Reform Judaism who were members of the United Synagogues of America (Quinn et al. 1982). The 2010 count also has direct reporting, but from an expanded four branches of Judaism (Conservative, Reconstructionist, Reform and Orthodox) (Grammich et al. 2012). In 1990 and 2000, however, the data collectors relied on the American Jewish Yearbook to identify adherents and the Jewish Almanac Yellow Pages and phone directories to identify congregations (Bradley et al. 1992; Jones et al. 2002).
- *Independent Churches* No efforts were made to count Independent Churches in 1980 and their measurement changed significantly between 2000 and 2010. In 1990 and 2000, the Megachurch Research Center gathered information on large, independent churches for the religion census by calling the churches via telephone, identifying about two million adherents in each year (Bradley et al. 1992; Jones et al. 2002). In 2010, a more exhaustive study using Internet listings was possible, identifying 10 million more adherents than in the 1990 and 2000 religion censuses (Grammich et al. 2012).
- *Old Order Amish* Adherents in 1980 and 1990 were estimated using an arbitrary congregational size based on the age of the church (Quinn et al. 1982; Bradley et al. 1992).^8^ In 2000, the data were supplied from the Mennonite publishing house and were more congregationally specific in estimation (Jones et al. 2002). For 2010, an exhaustive independent study was commissioned to conduct an accurate census using Amish settlement directories (Grammich et al. 2012).

In summary, longitudinal analyses specific to these groups is limited. Some of the caveats against overtime comparisons are negligible, depending on research questions and analytic plans.^9^ For instance, Catholic and LDS adherent data are comparable between 1980 and 2000, and the congregational data is comparable through 2010. Researchers who wish to include the United Methodists in their analyses must choose between the slightly less accurate county-level UMC adherent estimates for a full 1980–2010 comparison, or use the newer adherent reports that are limited to 2000 and 2010. Similarly, some comparisons can be made among the Amish between 1980 and 1990, the Jewish and Independent Church counts between 1990 and 2000, and many Orthodox groups can be compared between 2000 and 2010. We strongly advise against making any comparisons at all among Black Protestant groups or the Wesleyan Church.

### Missing Data

When merging the data, a third challenge is accounting for missing data in one or more of the collections. The vast majority of groups in the religion census have missing data in at least one of the datasets. Due to the expanding scope of the collections as well as individual group’s partial or non-participation in one or more years, the pattern of missing data in the religion census is diverse. Using two missing data strategies, we were able to estimate the missing data of 40 religious groups. Of these, 26 groups become available across all four datasets, 10 groups are limited to a 2 or 3-year comparison, and the remaining four groups are included in an aggregate grouping (as noted in the merger and schism section). These strategies increased group participation^10^ across all four datasets from 48 to 76, and from 92 to 111 in a 2-year comparison between 2000 and 2010. Appendix B lists all the affected groups and their new estimates.

The first missing data strategy uses simple linear interpolation and extrapolation to estimate the number of adherents and congregations among 23 groups who are missing both data types in only one of the four datasets. We limited our estimates to these 23 groups in the interest of retaining as many groups as possible without compromising the reliability and validity of the counts. Linear interpolation estimates the values between two points in time, and extrapolation estimates values beyond them, by continuing the line formed between the points.^11^ An interpolation and extrapolation strategy is commonly used in economics research to fill in missing data for entire variable values. Past research has used interpolation/extrapolation with the religion census data to estimate values of total adherence in the years between and beyond the decennial datasets (Hillary and Hui 2009; Dyreng et al. 2012; Boone et al. 2013). The main assumption in this strategy is that adherent and congregation size has a linear relationship with time. This is the case for the vast majority of groups in the religion census data, and studies on church growth and decline usually observe a linear relationship with time (Finke and Stark 1986; Blau et al. 1993; Hadaway and Roozen 1993; Marcum 2017). Because we limited our use of the interpolation/extrapolation strategy to select groups with nearly complete data, we believe that the estimates are reasonable within the assumption of linear growth/decline.^12^ We estimated the counts of 14 groups via extrapolation for 1980 or 2010, and another nine groups had their data estimated via interpolation for either 1990 or 2000, adding a cumulative 1.5 million adherents to the longitudinal file (see Table 3).^13^

**Table 3:** Total adherents added using missing data estimations

	1980	1990	2000	2010	Total
Strategy 1	718,838	424,061	75,334	312,999	1,531,232
Strategy 2			86,081	837,524	923,605
Total	718,838	424,061	161,415	1,150,523	2,454,837

Totals are rounded; individual estimates are kept un-rounded

The second missing data strategy was applied to 17 groups. These groups had congregation counts but did not have adherent counts in either the 2000 or 2010 dataset. We estimated adherent counts in the missing year by multiplying their congregation counts by the observed adherent-to-congregation ratios (i.e. congregation size) within each county in the other year. We focused on the 2000–2010 time period because the majority of adherent-only missing data occurs in these 2 years. This focus also improves the representation of Eastern Orthodox groups, for whom 2000 and 2010 are the only years when their measurement is comparable.^14^ Estimations of adherents based on congregation-size have been used in the religion census data collections before, such as the 100 membership size applied to some Black Protestant congregations in the 2010 dataset (Grammich et al. 2012). Such assigned congregation sizes, however, are relatively arbitrary and applied to counties of all characteristics. Our strategy improves on past estimations by using data from another year to inform the congregation size unique to each county, thereby capturing a more accurate picture of each group’s geographic distribution and the addition/subtraction of adherents as congregations open or close. We used a few variations of the congregation-based strategy depending on the needs of the group. The adherents of three Eastern Orthodox groups were estimated in 2000 based on their adherent-to-congregation ratios in 2010.^15^ Another 10 groups were missing adherents in 2010, and so we used their congregation size from 2000 as the multiplier.^16^ For counties where there were congregations in 1 year, but not another, we assigned a conservative “small church” size of 40 adherents per congregation in that county.

We also adjusted our congregation-size strategy to estimate the adherent counts of the four groups affected by mergers and schisms. These four groups are: the Serbian Orthodox Church in the USA (New Gracanica Metropolitanate), Friends Unaffiliated Local Meetings, the Anglican Church in North America (ACNA), and the North American Lutheran Church (NALC). In Appendix B, a value of “M” indicates that these groups’ data are merged with another group for that particular year. These four groups lack not only adherent counts in both 2000 and 2010, but their congregation counts are also missing in one of the years. To address the data limitations, we drew the adherent to congregation ratios from larger religious groups with which they share history and ideology (e.g. other Eastern Orthodox or Friends groups). In doing so, we are assuming that the groups with missing data have distributions similar to other groups with whom they share a theology and history, which is generally supported in the literature on religious regions (Stump 1998; Bauer 2012). For the Serbian Orthodox Church, we used the ratio of all other Orthodox groups that participated in the 2000 dataset and multiplied the ratio by the number of Serbian congregations. For the Friends Unaffiliated Local Meetings we used the ratio from all other participating Friends groups in 2010. For the ACNA and NALC, we used all Evangelical Protestants.^17^ Both the ACNA and the NALC have national-level statistics about their congregation and membership size on their official websites, which provide additional insight into the size that the congregations of each group tend to be. Their national-level congregational size was weighted by the geographical representation of other Evangelical Protestants in the 2010 dataset.^18^ The congregation-size estimate recovers nearly 1 million adherents across 2000 and 2010, and both strategies together add nearly 2.5 million across all 4 years of data (Table 3).

The two missing data strategies are not without caveats. The congregation-size strategy assumes that the average congregation size within the county for each group is the same over two decades. In reality, it is foreseeable that the addition or loss of a congregation could alter the average congregation size in a county, and recent evidence suggests that smaller congregations are more likely to close than large ones, which are becoming even larger in recent decades (Chaves 2017). These factors indicate that change in average congregation size between decades does occur, and our approach masks the co-existence of small and large congregations that open or close at different rates within the same county. Among the schism/merger groups, we further assumed that the congregation size and/or distribution of the group matched that of ideologically similar religious groups, although there are likely counties where this is not the case. Our interpolation and extrapolation techniques also provide relatively simplistic estimates of missing data, where the only relationship considered is a linear one with time. Extrapolation also cannot predict future geographic expansion, but this problem is limited to only two groups for whom we extrapolated values in the 2010 dataset. The simplicity of our missing data methods, however, has future value; it is relatively easy for data users to update them using the 2020 religion census when it becomes available. We recommend that researchers use the missing data estimates within larger religious group traditions or families, such as measuring the size of all Evangelical or Friends groups, rather than to accurately represent the growth or decline of the specific religious group. Overall, we believe our estimates improve the data by increasing group representation and allowing for the adherent counts of groups involved in mergers or schisms to be included in aggregate groupings appropriate for longitudinal analyses.

### Standardizing Areal Units

Whereas the first three challenges addressed how to offer comparable counts of congregations and adherents over time, the final challenge is concerned with the unit of analysis. While no changes in state boundaries have occurred in many decades, a total of 92 counties are affected by changes between 1980 and 2010 that are problematic for data users. For instance, the creation of a county results in the re-allocation of other counties’ population into the new county, while the dissolution of a county results in its population being allocated to one or more surrounding counties. The 1980 and 1990 religion census datasets also are distinct from the 2000 and 2010 datasets in how they treat independent cities in Virginia. In 1980 and 1990, many (but not all) of the religious group counts in independent cities were attributed to their surrounding counties, making it appear that the independent cities have missing data and that the surrounding counties had more adherents than actual (Quinn et al. 1982; Bradley et al. 1992). In 2000 and 2010, the counts in independent cities have adherent/congregation counts separate from their surrounding counties. This problem makes it impossible to compare the counts in Virginia independent cities and surrounding counties from 1980/1990 to 2000/2010 without aggregating them.

To keep counties consistent across all four waves, we created aggregate county units to standardize their boundaries. For instance, the creation of Broomfield County in Colorado in 2001 took territory from four surrounding counties. We therefore merged all five of the counties for 1980–2010 to maintain consistent boundaries. Likewise, counties that were dissolved over the time period were combined with the counties they dissolved into for the years prior to their dissolution. South Boston City and Clifton Forge City counties in Virginia were among the counties in the U.S. that dissolved over the time period, as are several territories in Alaska. Secondly, we addressed the problem of the independent cities in Virginia, which affected 61 county and county-equivalents. We merged the independent cities with their ‘parent’ counties in 2000 and 2010, just as they appear in 1980 and 1990. Each of the county mergers are detailed in Appendix C. In the original 2010 dataset, there are 3149 counties. After implementing the necessary county mergers between 1980 and 2010, there are 3096 counties and county-equivalents in the new longitudinal file.^19^

## Summary of Improvements and Remaining Limitations

Table 4 summarizes the consequences of our many adjustments.^20^ Along with making the data more comparable overtime, the adjustments also sharply increased the number of adherents that could be included from each collection in the final merged files. The most substantial increases were for the white Protestant groups. If we were to use the religion census datasets without any adjustments, several Protestant groups would be omitted due to schisms, mergers, aggregations, measurement changes and missing data. Accounting for schisms, mergers and other group aggregations allows for the full inclusion of the Episcopal Church, the Evangelical Lutheran Churches in America, several additional Mennonite groups, all of the Friends groups and the Moravian Church in America (see Table 1). As shown in Table 4, adjusting for mergers and schisms adds between six and nine million additional adherents in each year of the dataset, particularly benefiting the Mainline aggregate count. Including the United Methodist Church after adjusting for their measurement change also greatly increases Mainline adherents by about 10 million each year. Including the missing data estimates (net those involved in the mergers and schisms) adds approximately 1.5 million more adherents each year. We also can see the benefit of the county mergers in the last section of Table 4. By including the merged county units, we added between two and three million Protestant adherents to the dataset. Taken altogether, the changes allowed for the more reliable inclusion of an additional 20–23 million Protestant adherents in each year.

**Table 4:** Summary of adjustments

	1980	1990	2000	2010
*Unadjusted* ^*a*^				
Total	84,027,167	92,453,003	102,537,360	98,410,969
Mainline	9,544,748	8,741,510	7,874,053	6,302,438
Evangelical	27,622,578	30,997,118	33,750,805	34,313,392
Adjustments:				
*Schisms, mergers, and aggregations*				
Total	92,385,413	100,296,147	110,088,921	105,178,553
Mainline	17,739,657	16,392,238	15,257,697	12,930,582
Evangelical	27,785,915	31,189,534	33,918,722	34,452,832
*United Methodist* ^*b*^				
Total	103,619,661	111,081,456	120,162,319	114,196,449
Mainline	28,973,905	27,177,547	25,331,095	21,948,478
Evangelical	27,785,915	31,189,534	33,918,722	34,452,832
*Missing data*				
Total	105,070,105	112,545,476	121,691,230	115,769,243
Mainline	29,048,762	27,234,623	25,370,665	21,974,128
Evangelical	29,161,502	32,596,478	35,408,063	35,999,976
*County merges*				
Total	107,266,898	114,820,546	124,477,132	118,603,783
Mainline	29,764,906	27,931,687	26,027,135	22,569,004
Evangelical	29,843,554	33,312,668	36,232,257	36,895,286

^a^Only includes groups with consistent adherent measures in all 4 years

^b^Uses the “old” county-level adherent estimate

Despite these improvements, limitations in utilizing all 4 years of data for longitudinal analyses remain. We did not estimate adherent and congregation counts of all the groups who had missing data, and not all groups involved in schisms are present in the years following their founding. The most significant limitations, however, are the unamendable measurement changes among some of the largest religious groups. Adherent comparisons across all 4 years are mostly limited to predominantly white Mainline and Evangelical Christian denominations. If the analyses are limited to fewer years of data or to congregational counts, however, the new longitudinal files still can be used to study additional groups, such as Catholics, Jews, Mormons, or some of the Orthodox Christian groups (see Table 2). Unfortunately, the historically African-American denominations are not fully represented in any of the collections. Limitations remain, but the new longitudinal files offer important improvements for using the religions censuses for overtime research.

## Conclusion

The decennial religious censuses now collected by the Association of Statisticians of American Religious Bodies have been used extensively by researchers; but the complexities of merging the files have prevented most from using the files for explaining religious change over time. Schisms and mergers, changing geographical boundaries, modified methods of reporting and various sources of missing data have posed challenges that made the merging both complex and time consuming. Some of the challenges required demographic training, others required an extensive knowledge of the specific groups being studied. Addressing these challenges required both expertise and time.

We have made several changes to the 1980, 1990, 2000 and 2010 religion census datasets in order to address challenges associated with merging the data collections into a longitudinal file. These changes included alterations to variable names, the creation of new combined groups to correct for schisms and mergers, introducing alternative counts of the United Methodist Church, providing estimates for missing data and merging county units in the county-level file. Collectively, these changes produced new longitudinal files that increase religious group representation, reduce bias from missing data, improve the file’s ease of use and are readily accessible from theARDA.com.

We have issued several warnings on the limitations of the data and on which groups can be meaningfully compared over time, but this shouldn’t distract from the many opportunities the merged files offer. For a core group of 70 religious groups,^21^ meaningful comparisons among adherents and congregations now can be made using all four collections. For many of the remaining groups, comparable data is now available for at least two points in time. This allows researchers to explore how groups change in size and geography over time and how these changes are related to other social, demographic and economic changes. Overall, we believe that our efforts greatly improve the quality of the data by reducing missing data and making the data more comparable over time, and will simplify the process of accessing and using a merged file for all users.

## Footnotes

1 Both files can be downloaded from theARDA.com at: http://www.thearda.com/Archive/Files/Descriptions/RCMSMGCY.asp and http://www.thearda.com/Archive/Files/Descriptions/RCMSMGST.asp.

2 Reported Church Members × [Total Population/(Total Population − Children 13 years and under)]. In the 2010 collection, 85 groups reported their own adherents while an additional 58 groups reported members and had adherents estimated by data collectors using the county-level multiplier (Grammich et al. 2012).

3 The 1980 documentation identifies 111 groups because the American Lutheran Church, Lutheran Church in America, and Association of Evangelical Lutheran Churches reported separately in the publication, but were merged in the stand-alone 1980 dataset available on the ARDA website.

4 The organizations helping to fund one or more of the collection have included: the Lilly Endowment Inc., John Templeton Foundation, National Council of the Churches of Christ in the U.S.A. (New York), Association of Statisticians of American Religious Bodies, Glenmary Research Center, Glenmary Home Missioners,  Aid Association for Lutherans, Southern Baptist Convention, Lutheran Council in the U.S.A., African Methodist Episcopal Zion Church, Church of the Nazarene, American Baptist Churches in the USA, National Association of Free Will Baptists, Presbyterian Church (U.S.A.), United Church of Christ, Wisconsin Evangelical Lutheran Synod and Evangelical Lutheran Church in America.

5 The merged file also includes a variable that flags whether each case is included elsewhere in a combined group or is itself a combined group.

6 The omission of the AOB from SBC enumerations in the 1990 and subsequent collections is unlikely to be associated with significant bias. In 2010, there were 124 AOB congregations compared to 50,816 congregations in the SBC.

7 There are additional non-Christian groups (i.e. Buddhist, Jain, Muslim, and Hindu) whose data were collected by different independent PIs or with differing procedures between years, and only have counts for 2000 and 2010. We do not discuss those groups here due to their limited participation, but we do include notes about their measurement in Appendix A. More detailed descriptions of the methodologies used to count all groups in each collection are available in the appendices of the religion censuses’ data publications (Quinn et al. 1982; Bradley et al. 1992; Jones et al. 2002; Grammich et al. 2012).

8 For the 1990 collection, determined as 150 per congregation for settlements established before 1987 and those established in 1987 and 1988 were assigned 100 adherents per congregation. Settlements established in 1989 and 1990 had 50 per congregation (Bradley et al. 1992).

9 We include a flag variable in the merged file to identify groups with measurement problems that could prevent over-time comparisons.

10 Full group participation is reserved for groups with both adherent and congregation data. This count includes groups with changes in measurement (e.g. Catholics).

11 We used the “ipolate” command with the epolate option in Stata 14, which uses the following general formula: $y = \frac{{y_{1} - y_{0} }}{{x_{1} - x_{0} }}(x - x_{0} ) + y_{0}.$

12 For some groups, the aggregate count does not appear linear. This is because the formula is applied at the county level. The group may be in decline in some counties, but growing in others, resulting in an overall shift in size contrary to expectations.

13 In the merged file, a flag variable identifies the counts that are estimated using each missing data method.

14 There are 11 groups that have congregation counts but are missing adherents in 1990. We decided not to estimate these groups for two reasons: 1) they do not have sufficient participation in 2000 and 2010 to allow comparisons beyond 1990, or 2) they are an Eastern Orthodox group for whom measurement changes in 2000 disallow comparisons before and after 2000.

15 Adherents in 2000 = (Adherents in 2010/Congregations in 2010) × Congregations in 2000.

16 Adherents in 2010 = (Adherents in 2000/Congregations in 2000) × Congregations in 2010.

17 We chose Evangelical Protestants as the larger category for the ACNA and NALC because the groups are more conservative than their Mainline ‘parent’ denominations, and also because the Evangelical adjustment produced estimates closer to each group’s national membership than the Mainline adjustment would have. Evangelical Protestants in the 2010 dataset were identified using RELTRAD categorizations as a guide (Steensland et al. 2000).

18 We used the following formula: Missing Group Adherents = Group’s National Congregation Size × (County Evangelical Protestant Congregation Size/National Evangelical Protestant Congregation Size) × Group Congregations. The national congregation size for each group came from their official statistical reports available online. The ACNA’s most recent online report (Anglican Church in North America 2017) identified 116 adherents per congregation; we used a size of 100 as a more conservative estimate. The national congregation size for the NALC was 352 in 2015 (North American Lutheran Church 2016); we used a size of 300 as a more conservative estimate.

19 The longitudinal file is not available with de-aggregated counties. Aggregated county counts were necessary to calculate missing data and could not be deconstructed afterwards without additional bias. Researchers may contact the authors to inquire about altering the data to accommodate fewer aggregations (e.g. examining only 2000–2010 data doesn’t require Virginia Independent City aggregations).

20 Mainline and Evangelical categorization was based on the RELTRAD specification detailed by Steensland et al.(2000). Appendix A identifies the RELTRAD category assigned to each group and a RELTRAD variable is also included in the longitudinal file.

21 Six religious groups provided both adherent and congregation data in all 4 years, but are excluded from this count due to measurement changes in either adherents or congregations. The excluded groups are: Roman Catholics, Latter-Day Saints, Southern Baptists, Old Order Amish, the Armenian Apostolic Church of America (Eastern Prelancy), and Archdiocese of the Syrian Orthodox Church of Antioch.
